# Exploring Action Items to Address Resident Mistreatment through an Educational Workshop

**DOI:** 10.5811/westjem.2019.9.44253

**Published:** 2019-12-09

**Authors:** Max Griffith, Michael J. Clery, Butch Humbert, J. Michael Joyce, Marcia Perry, Robin R. Hemphill, Sally A. Santen

**Affiliations:** * Michigan Medicine/St. Joseph Mercy Ann Arbor, Department of Emergency Medicine, Ann Arbor, Michigan; † Emory University School of Medicine, Grady Memorial Hospital, Department of Emergency Medicine, Atlanta, Georgia; ‡ Indiana University School of Medicine, Department of Emergency Medicine, Indianapolis, Indiana; § Virginia Commonwealth University School of Medicine, Department of Emergency Medicine, Richmond, Virginia; ¶ Michigan Medicine, Department of Emergency Medicine, Ann Arbor, Michigan

## Abstract

Mistreatment of trainees is common in the clinical learning environment. Resident mistreatment is less frequently tracked than medical student mistreatment, but data suggest mistreatment remains prevalent at the resident level. To address resident mistreatment, the authors developed an Educational Advance to engage emergency medicine residents and faculty in understanding and improving their learning environment. The authors designed a small-group session with the following goals: 1) Develop a shared understanding of mistreatment and its magnitude; 2) Recognize the prevalence of resident mistreatment data and identify the most common types of mistreatment; 3) Relate study findings to personal or institutional experiences; and 4) Generate strategies for combating mistreatment and strengthening the clinical learning environment at their home institutions. Design was a combination of presentation, small group discussion, and facilitated discussion. Results were presented to participants from a previously administered survey of resident mistreatment. Public humiliation and sexist remarks were the most commonly reported forms. Faculty were the most frequent perpetrators, followed by residents and nurses. A majority of respondents who experienced mistreatment did not report the incident. Session participants were then asked to brainstorm strategies to combat mistreatment. Participants rated the session as effective in raising awareness about resident mistreatment and helping departments develop methods to improve the learning environment. Action items proposed by the group included coaching residents about how to respond to mistreatment, displaying signage in support of a positive learning environment, zero tolerance for mistreatment, clear instructions for reporting, and intentionality training to improve behavior.

## BACKGROUND

Mistreatment in medical education is common;^1^,^2^ however most reporting to date has focused on mistreatment experienced by medical students.^3^,^4^ Studies have linked medical student mistreatment with increased rates of burnout,^2^ and symptoms of post-traumatic stress.^5^ In addition to its effects on learners’ psyches, mistreatment in the medical learning environment is troubling as it may contribute to poor outcomes for patients.^6^

The American Association of Medical Colleges (AAMC) Graduation Questionnaire (GQ) has tracked medical student mistreatment since it first included questions about mistreatment in 1991.^1^ The GQ asks about experiences with sixteen mistreatment behaviors, such as public humiliation, discriminatory comments based on race, gender, ethnicity or sexual orientation, and being threatened with physical harm.^7^ There is no comparable tool for tracking rates of resident mistreatment. The existing literature on mistreatment experienced by residents is sparse but suggests that issues with the learning environment persist throughout medical training.^8^,^9^

As trainees at teaching hospitals, residents’ professional identities are shaped by their medical learning environments.^10^ The learning environment has been conceptualized as a combination of personal, physical, social and cultural factors, which, when supportive, helps learners thrive, and when unsupportive, contributes to depression and burnout.^11^ Improving the clinical learning environment by reducing resident mistreatment is an important goal for the wellbeing of the next generation of physicians, and by proxy, their patients. To address resident mistreatment, we developed an Educational Advance to engage emergency medicine (EM) residents and faculty in understanding and improving their learning environment.

## OBJECTIVES

We designed a small-group session using an approach based on the six-step approach to curricular development described by Kern et al which includes problem identification and general needs assessment, needs assessment for targeted learners, goals and objectives, educational strategies, implementation, and evaluation/feedback.^12^ A general needs assessment had previously been conducted in the form of a survey about resident mistreatment at three institutions demonstrating a high rate of mistreatment.^13^

For a second needs assessment for targeted learners – in this case EM residents, faculty and staff – we separated out mistreatment data as reported by EM residents only. We found that the rates and types of mistreatment reported by EM residents were generally similar to the combined data from residents across all specialties.

We identified the following goals and objectives for this session: 1) Develop a shared understanding of mistreatment and its magnitude; 2) Recognize the prevalence of resident mistreatment and identify the most common types of mistreatment; 3) Relate study findings to personal or institutional experiences; 4) Generate strategies for combating mistreatment and strengthening the clinical learning environment at their home institutions.

As part of a quality improvement project, these sessions were assigned Institutional Review Board “not regulated” status.

## CURRICULAR DESIGN

For educational strategy, a didactic format was chosen to provide background on the definition and scope of resident mistreatment, as well as an interactive small group component with facilitated discussion to draw on the diverse perspectives of participants. The conceptual framework was constructivist, with each participant building their own understanding from personal experience and discussion with others.

### Section 1

As an introduction, in order to develop shared understanding of mistreatment, participants were asked about past experiences with mistreatment. Responses were wide-ranging, from the perceived disrespect of referring to a resident by first name in front of patients rather than by their title of doctor, to publicly berating a resident for failing a line placement, accusing them of incompetence and blaming them for the patient’s poor outcome in front of the care team and patient family. This conversation established that mistreatment may be blatant or subtle, is subjective, and likely depends on the observer’s past experiences with discrimination or marginalization, as well as the power dynamics between the involved individuals.

The introduction was followed by presentation of data from a previous study which surveyed residents across multiple specialties at three institutions.^13^ The survey queried residents whether they had experienced various categories of mistreatment, with options similar to those found in the AAMC GQ. Residents were also asked whether or not they reported the mistreatment, their reasons for not reporting, and who the perpetrators of mistreatment were (e.g. faculty, other residents, or nursing staff). Public humiliation and sexist remarks/names were the most commonly reported forms of mistreatment. Additionally, residents reported faculty were the most frequent perpetrators of mistreatment, followed by other residents, and nurses. A minority of respondents who experienced mistreatment reported the incident to their institution or program. Reasons for not reporting were “Did not seem important enough,” “I did not think anything would be done about it,” “I resolved the issue myself,” and “I did not know what to do.” Following the presentation, participants engaged in small group discussions about participants’ personal or witnessed experiences with resident mistreatment.

### Section 2

The session facilitators provided a brief review of institutional practices for addressing resident mistreatment, outlined in Table 1. Participants were then asked to brainstorm and share strategies to combat mistreatment so that together they might develop strategies to take back to their programs.

Implementation involved identifying appropriate settings for this session to take place. This session was facilitated twice in two separate settings: as part of the weekly educational conference for an EM residency program and as a didactic at the Society for Academic Emergency Medicine annual meeting in 2019.

Evaluation and feedback were gathered through an electronic evaluation form that all participants were encouraged to complete.

## IMPACT/EFFECTIVENESS

At the conclusion of each of the two sessions, participants were asked to complete an electronic evaluation of the sessions’ effectiveness. Twenty-five participants completed evaluations (9/10 at SAEM and 16/28 at the residency conference) for an overall response rate of 71%. Respondents included 13 residents and 12 attendings/fellows. One hundred percent responded “yes” to the question, “Was this workshop effective in raising awareness of the problem of resident mistreatment?” Similarly, 100% responded yes to the question, “Did this workshop help your department to come up with ways to improve the learning environment?”

Participants were asked whether they would make any changes to the way they approached learners, trainees or other staff in the learning environment. Examples of free responses were wide-ranging and included, “Encourage [residents] to provide feedback to others if they feel there is an improper interaction,” “introspection of my own handling of resident communication,” “I will be more respectful about privacy when providing evals,” and “Will consider signage and emphasizing the chain of command”

The institutional interventions that were proposed to address resident mistreatment approach the issue from multiple standpoints: personal (coaching residents), physical (supporting signage), social (training), and cultural (zero tolerance policy). Further research is required to determine whether any of these proposed interventions would reduce rates of resident mistreatment, though the variety of approaches offers multiple avenues to address a common problem.

Resident and faculty participants also included feedback for how to improve future iterations of this session. Ideas included providing more specific tools for how to combat mistreatment, as well as incorporating interactive activities to help participants build skills to address mistreatment.

Session facilitators wrote down action items that emerged from the group discussion on strategies to combat mistreatment, detailed below. We provide the interventions in this Educational Advance to help others who might use a similar discussion session to begin the conversation about how to address mistreatment at their own programs.

A zero-tolerance policy for episodes of mistreatment was identified as essential. Given that residents under-report mistreatment due to concern that nothing will be done, it is important to demonstrate that there are real repercussions for those who mistreat residents. This should be placed in departmental policy as well as faculty manuals. The emergency department or other clinical setting may display a prominent sign stating abusive behavior will not be tolerated. If anyone displays abusive behavior to the resident, she may point out the sign as a clear and official reminder. This is a visible reminder to all faculty, staff and residents, but also to patients and families (a significant source of mistreatment beyond the scope of this paper).

In order to effectively address resident concerns about mistreatment, instances of mistreatment must be consistently and thoroughly documented. Session participants recommended instructing residents to document very clearly what was said or done, and by whom, in order to equip the institution with the necessary information to make an intervention. This must be done in a manner that feels safe for the resident and, depending on who is involved, may require using resources outside of the department. Policies should be in place to define when this is appropriate. Similarly, some residents had reported not knowing what to do with their concerns about mistreatment. For this reason, we highlighted the importance of detailing an explicit chain of command so that residents know who to approach with concerns. In addition, any institutional mechanisms, online reporting or ombudsperson should also be publicized so residents are aware of their existence and how you can use them to report mistreatment.

Faculty can coach residents about how to respond to mistreatment. As an example, a resident was publicly berated by an attending for failing a line placement. It was emphasized that residency leadership has the responsibility to defend the resident after the fact, and it is also important that the resident displays professionalism in the moment. Feedback and expectations should be provided to the involved faculty, but repeated behaviors must be addressed.

Finally, participants recognized the potential to develop intentionality through training. Microaggressions, increasingly recognized as a form of discrimination in the medical workplace,^14^ may be unintentional or stem from lack of awareness on the part of the perpetrator. Multiple discussants identified systems their programs had developed to reduce, or at least acknowledge, microaggressions in the clinical environment. One example was an institutionally-supported code word that anyone could speak when they perceived a microaggression, empowering people to speak out. Another example involved an acronym that was taught to encourage mindfulness about interpersonal interactions. Based on our discussions, the need for training on self-reflection and thoughtful communication cannot be understated.

A limitation to this study is the level of impact. Participants reported they would change their approach to interactions with others as a result of this workshop; however, this reflects a hypothetical change in behavior which is subject to desirability bias. Further studies might explore perceived changes in program culture following the workshop. Participants were also self-selected, suggesting they already had an interest in tackling this problem. Future workshops will need to target all members of the learning environment. While we gathered data about participants status as resident or fellow/attending, we did not ask about demographic factors such as gender, ethnicity or age. With more participants, it would be informative to analyze for differences according to demographic groups. Additionally, participants responded that this session helped them come up with departmental solutions to improve the learning environment, but we have no information about how many ideas generated during this session were followed by departmental action or whether these actions were effective. It is our hope that this or similar sessions will provide the foundations for future interventions which will be measured and reported on.

Mistreatment of residents is common and detrimental to resident training and may have a negative impact on healthcare team dynamics and patient care. Residencies have a responsibility to foster a productive learning environment though there are many possible approaches. In conclusion, through two small group sessions, we were able to develop a better shared understanding of mistreatment and generate a list of action items to take on the issue. We have described an interactive educational session which can be applied in other settings to generate ideas – a first step to addressing this problem for residency programs.

## Figures and Tables

**Table 1:** Practices for addressing mistreatment at the institutional level with tips for successful implementation.

Systems for reporting all instances of mistreatment
Conduct needs assessment to quantify the problem and identify problem areas
Anonymity may facilitate reporting
Unified messaging defining mistreatment & behavioral expectations
Avoid ambiguity with a single, clear message backed by unequivocal action
Communication and behavioral training for residents and faculty
Increase self-awareness through role playing and simulation with feedback
Establish positive culture
Provide well-defined professionalism policies/procedures
Include domains of mistreatment on annual evaluations
Introduce concepts during onboarding, reinforce periodically
